# Next generation sequencing of viral RNA genomes

**DOI:** 10.1186/1471-2164-14-444

**Published:** 2013-07-04

**Authors:** Denise A Marston, Lorraine M McElhinney, Richard J Ellis, Daniel L Horton, Emma L Wise, Stacey L Leech, Dan David, Xavier de Lamballerie, Anthony R Fooks

**Affiliations:** 1 Wildlife Zoonoses & Vector-Borne Diseases Research Group, Animal Health & Veterinary Laboratories Agency (AHVLA), New Haw, Addlestone, Surrey KT15 3NB, UK; 2 Central Sequencing Unit, Animal Health & Veterinary Laboratories Agency (AHVLA), New Haw, Addlestone, Surrey KT15 3NB, UK; 3 National Consortium for Zoonosis Research, Leahurst, Neston, Wirral, UK; 4 Rabies Laboratory, Kimron Veterinary Institute, Bet Dagan 50250, Israel; 5 Aix Marseille Univ, IRD French Institute of Research for Development, EHESP French School of Public Health, UMR_D 190, “Emergence de Pathologies Virales”, 13005 Marseille, France

**Keywords:** Next generation sequencing, Pyrosequencing, Lyssavirus, Genome, RNA, Virus

## Abstract

**Background:**

With the advent of Next Generation Sequencing (NGS) technologies, the ability to generate large amounts of sequence data has revolutionized the genomics field. Most RNA viruses have relatively small genomes in comparison to other organisms and as such, would appear to be an obvious success story for the use of NGS technologies. However, due to the relatively low abundance of viral RNA in relation to host RNA, RNA viruses have proved relatively difficult to sequence using NGS technologies. Here we detail a simple, robust methodology, without the use of ultra-centrifugation, filtration or viral enrichment protocols, to prepare RNA from diagnostic clinical tissue samples, cell monolayers and tissue culture supernatant, for subsequent sequencing on the Roche 454 platform.

**Results:**

As representative RNA viruses, full genome sequence was successfully obtained from known lyssaviruses belonging to recognized species and a novel lyssavirus species using these protocols and assembling the reads using *de novo* algorithms. Furthermore, genome sequences were generated from considerably less than 200 ng RNA, indicating that manufacturers’ minimum template guidance is conservative. In addition to obtaining genome consensus sequence, a high proportion of SNPs (Single Nucleotide Polymorphisms) were identified in the majority of samples analyzed.

**Conclusions:**

The approaches reported clearly facilitate successful full genome lyssavirus sequencing and can be universally applied to discovering and obtaining consensus genome sequences of RNA viruses from a variety of sources.

## Background

RNA viruses have small, simple genomes, which have a high level of diversity, due to the low-fidelity viral polymerase used for replication. Traditionally, due to their small genomes, ‘genome-walking’ was used to obtain a reference sequence, from which primer pairs can be designed for down-stream use on similar viruses [1-6]. However, this methodology can take a large amount of time, effort and expense, and be cumbersome, not least because of the variation within virus species, which results in the need to redesign primer pairs and frequent re-optimization of conditions. In addition to these optimization issues, every novel virus discovered will require ‘genome walking’. Moreover, the introduction of PCR errors using Sanger-based sequencing is problematic, particularly when sequencing from cloned DNA. The use of high-fidelity enzymes and sequencing PCR products directly can overcome this issue in part, but errors occurring early in the amplification process will be sequenced incorrectly [7]. These problems can be avoided by utilizing Next Generation Sequencing (NGS), a high-throughput sequencing methodology which generates millions of sequences simultaneously from one sample [8]. Multiple platforms are available, the two commonly used are Roche 454 pyrosequencing (454 Life Science) and Illumina (Solexa) [9]. These platforms were initially developed and applied to mammalian and bacterial genomes, where Sanger methods were prohibitively expensive. Because viral genome sequencing is achievable using the traditional PCR/Sanger methods, the virology field has had less necessity to embrace this new technology. The current NGS assays are optimized for large bacterial or mammalian genomes, where nucleic acid is in abundance and obtaining the hundreds of nanograms of nucleic acid required, is relatively simple. Due to the low percentage of viral RNA in relation to host RNA in any given sample, obtaining the quantity of viral RNA required by the protocols can be problematic.

The advantages of NGS technologies in virology are numerous, and its use is becoming more commonplace [10], particularly to detect and characterize pathogens without prior knowledge of their existence, or in association with the sequelae/disease outcome, in primary or cultured material, without the requirement of specific primers [11,12]. Often however, the virus is at such low abundance in these samples that only one or two reads can be obtained, or at best, a number of unlinked contiguous sequences (contigs), for which traditional Sanger protocols are employed to complete the genome [13,14]. Conversely, known positive samples have been ‘deep sequenced’ using NGS technologies, for a plethora of uses including determining viral heterogeneity, or the effect of the immune system or pharmaceutical drugs on viruses [15-19]. These deep sequencing methodologies employ the use of viral PCR amplicons to obtain a depth of coverage sufficient to detect variants which occur down to 0.1% frequency. Between these two approaches, a methodology to obtain full genome sequences directly from known positive clinical specimens or cultured material, without the use of amplicons, viral enrichment or virus concentration is still lacking. To this end, we describe a broadly applicable approach to obtaining full genome sequences from clinical or cultured samples, which we have successfully applied to lyssavirus infected samples.

Lyssaviruses (family *Rhabdoviridae*) have negative-sense, single-stranded RNA genomes approximately 12 kb long. Traditionally, partial gene sequence (usually the N-gene) was utilized for viral speciation and phylogenetic analysis. However, limitations on the information obtained from these partial sequences and a requirement to use full genome sequence as part of the ICTV (International Committee for Taxonomy of Viruses) criteria to propose new lyssavirus species, in combination with recent advances in high-throughput sequencing, have resulted in an increase of full genome sequences available on Genbank. From the submission of the first full length lyssavirus genome sequence of RABV (prototype) in 1988 [20] to the completion of representative genomes from each of the established twelve lyssavirus species (RABV, LBV, MOKV, DUVV, EBLV-1, EBLV-2 and ABLV, ARAV, KHUV, IRKV, WCBV and SHIBV) in 2010, the commonly used methodology for obtaining the genomes was relatively unchanged, utilizing reverse transcription then either cloning the cDNA directly, or cloning amplified PCR products, and Sanger sequencing [2,5]. Of the most recently described lyssavirus species, BBLV [21] and IKOV [22], only the IKOV characterization employed NGS technologies to obtain the full genome sequence, pioneering the way forward to obtaining genome sequences without the requirement to design multiple primers necessary for genome walking.

This study describes the optimization of protocols, required for a variety of starting material, describing a robust, simple, reliable methodology to obtain full genome sequences from original clinical material and cultured samples, both from the cell monolayer and the supernatant. Furthermore, we investigate the SNPs (Single Nucleotide Polymorphisms) observed in the sequences obtained.

## Results

### Optimization of the extraction protocol for tissue samples

Two different extraction methodologies were compared to investigate the suitability of the RNA obtained. Three brain tissue samples (RV2772, RV2627 and RV2516) were extracted, using the same amount of starting material, either by TRIzol® extraction followed by isopropanol precipitation, or RNeasy® plus mini kit (see Table 1 for isolate details and Table 2 for details of extraction). Genomic DNA depletion was carried out on-column, either as a separate step for the TRIzol® extracted RNA, or as part of the RNA extraction procedure, using the RNeasy® plus mini kit. The RNA concentration from the RNeasy® kit extracted samples were significantly lower than the TRIzol® extracted equivalents (Table 2). The required 200 ng RNA (10 ng/μl), was not achieved for the RNeasy® kit samples, or indeed for the RV2772 TRIzol® extracted sample. For these samples the maximum RNA (20 μl) was used with varying success.

**Table 1:** Details of virus isolates analysed in this study

**Virus**	**Original reference**	**Host species**	**Origin**	**Year of isolation**	**GenBank no.**
**Lyssavirus RABV**					
RV2324	ISR-50	Dog	Israel	1950	KF154998
RV50	AZBAT 7453	Bat (*Eptesicus fuscus*)	US, Az	1975	JQ685956*
RV61	-	Human (canine strain)	UK (ex-India)	1987	KF154996
RV437	269	Raccoon Dog	Estonia	-	KF154997
RV2417	R16/08	Dog	UK (ex Sri Lanka)	2008	KF154999
RV2627	CASA 09/08	Cow (Canine strain)	Morocco	2009	KF155001
RV2772	Rab91/ D10-867	Dog	Tanzania	2010	KF155002
RV2516	23	Cow (Canine strain)	Iraq	2010	KF155000
**Lyssavirus EBLV-1**					
RV20	RA552	Bat (*Eptesicus serotinus*)	Denmark	1986	KF155003
**Lyssavirus EBLV-2**					
RV1787	603-04^†^	Bat (*Myotis Daubentonii)*	UK (Staines)	2004	KF155004
**Lyssavirus IKOV**					
RV2508	snp0971^#^	African Civet	Tanzania	2011	JX193798

* Published after sequence was obtained prior to this publication.

^†^ Sequence confirmed by Sanger sequencing from the bat brain material.

^#^Both brain and TCSN were sequenced. For analysis see Table 2.

- Data not available.

**Table 2:** Details of brain and BHK cultured samples, relating to the extraction method used and RNA concentration during preparation stages resulting in number of reads obtained and outcome of obtaining full viral genome sequence

**Virus**	**Sample origin**	**Extraction method**	**Concentration after RNA extraction (ng/μl)**	**Concentration after gDNA depletion (ng/μl)**	**Concentration RNA using Ribogreen**^**1 **^**(ng/μl)**	**Total N**^**o **^**of reads**	**N**^**o **^**of vial reads**	**% viral specific reads**	**Genome as single contig**	**De Novo detection**
**Brain tissue samples**^**†**^										
RV437	mouse	TRIzol®	5,404.4	410.77	121.77	282,370	1,489	0.53	Yes^a^	Yes
RV61	mouse	TRIzol®	3,362.8	ND	58.9	191,508^#^	1,810	0.95	Yes^c^	Yes
RV2417	Dog	TRIzol®	5,431.3	1799.21	57.78	96,014	1,488	1.55	Yes^b^	Yes
RV2508	African civet^	TRIzol®	6,537.4	66.55	28.18	139,841	626	0.45	Yes^a^	Yes
RV2627	Cow	TRIzol®	2,385.8	111.81	26.60	11,470	682	5.95	Yes	Yes
		RNeasy mini	N/A	4.99	0.95	239	2	0.84	No	No
RV2516	Cow	TRIzol®	1,891.2	45.84	11.09	154,068^#^	550	0.36	Yes^a^	Yes
		RNeasy mini	N/A	11.12	2.14	7,991	17	0.21	No	No
RV50	mouse	TRIzol®	2,186.2	18.76	7.64	76,568	293	0.38	Yes	Yes
RV2772	Dog	TRIzol®	1,833.9	3.27	1.50	305,110	391	0.13	Yes	Yes
		RNeasy mini	N/A	7.19	1.08	48,849	180	0.37	No	Yes
**BHK cultured virus samples**										
RV2324	cell pellet	TRIzol®	10,232.9	870.72	75.63	101,599	2,027	1.99	Yes	Yes
RV20 depleted	TCSN 100 ml	TRIzol® LS	3,732.6	2.8	0.52	2,537	1,054	41.55	Yes	Yes
RV20			Diluted to 1,000	ND	23.47	10,332	4,369	42.29	Yes	Yes
RV1787 depleted	TCSN 100 ml	TRIzol® LS	4,603	37.87	11.56	8,162	53	0.65	No	Yes
RV1787			Diluted to 1,000	ND	119.47	6,861	87	1.27	No	Yes
RV2508 cells	TCSN 250 μl	TRIzol® LS	129.44	ND	11.45	14,587^#^	223	1.529	No*	Yes
RV2508 no cells			127.05	ND	0.46	326	42	12.88	No*	Yes
RV2508 high titre			112.5	ND	1.31	1,132	12	1.06	No*	Yes

*TCSN* tissue culture supernatant; N/A – Not Applicable as DNAse treatment is incorporated in the RNA extraction protocol, therefore no concentration before gDNA depletion is available; ND - Not Done as gDNA was not depleted from these samples; ^1^This column contains the RNA concentration after rRNA depletion for all samples, except RV20, RV1787 and RV2508 TCSN where no rRNA depletion was undertaken. ^†^brain either from host, or from one passage in a mouse, *combining these reads resulted in a single genome contig; # combined results from two 454 runs of same library; ^ Stored in RNAlater; ^a^ 3’UTR not represented; ^b^ 5’UTR not represented; ^c^ 3’ and 5’ UTR not represented.

Brain tissue samples ordered by concentration after the depletion process. Where rRNA depletion was not undertaken (RV20, RV1787 and RV2508 TCSN), the concentration values in the Ribogreen column are directly comparable to the RNA extraction concentration.

The number of total reads and viral reads obtained for the RNeasy® kit samples were lower in comparison to the TRIzol® extracted RNA, most likely due to the difference in total RNA available for these samples. On the whole, viral RNA was not enriched by the RNeasy® kit, as the percentage of viral reads was less for RV2627 and RV2516, although for RV2772 there was a slight increase in viral specific reads. Without exception, none of the RNeasy® kit extracted sample reads were sufficient to obtain a single consensus sequence, due to the low number of viral reads obtained. Furthermore, *de novo* assembly on two of the three samples (RV2516 and RV2627) failed to align viral reads into contigs for further analysis resulting in only host contigs being identified (Table 2).

### Analysis of depletion methodology

Regardless of the originating sample (brain tissue, cell monolayer, tissue culture supernatant) the concentration of the TRIzol® extracted RNA after gDNA depletion was significantly less than the original extract RNA sample (Table 2). The largest reduction was for RV2772 where RNA at 1,833 ng/μl was depleted to 3.27 ng/μl (600-fold reduction) after removal of genomic DNA. Interestingly, this sample was part of a cohort of samples that were highly degraded upon receipt, therefore the majority of RNA had already been degraded. Otherwise a reduction of concentration between 3-fold and 100-fold was observed (Table 2). The subsequent depletion of rRNA resulted in a more conservative fold change of concentration between 30-fold (RV2417) and 2-fold (RV2772 and RV2508).

We investigated the requirement to deplete gDNA and rRNA in cultured viral samples after RNA extraction, since the amount of cellular material would be minimal in these supernatant preparations. Comparison at the RiboGreen stage determined that RV20 and RV1787 depleted samples are 45-fold and 10-fold less than the RV20 and RV1787 non-depleted RNA samples respectively. Indeed, for RV20 the total amount of RNA was too low to obtain 200 ng RNA for fragmentation. The virus titer of RV1787 and RV20 has been calculated previously [23,24] with RV1787 (EBLV-2) approximately 1 log lower than RV20, therefore the difference in the percentage of viral reads is likely to be a reflection of this. Despite the marked difference between the percentage of viral reads of RV20 and RV1787, the difference within samples regarding whether the RNA was depleted or not, is not so obvious. Indeed, for both samples, the RNA sequenced directly without depletion provided more viral-specific reads.

The success of the methodology for both tissue material and cell cultured material, particularly the ability to detect viral sequence after *de novo* assembly, is illustrated in Table 2. Apart from the column-extracted samples, all sequences obtained were sufficient to obtain viral specific reads either as a single contig, or a number of contigs, which subsequently can be identified by a BLAST search.

### Depth of coverage analysis

**Figure 1:**
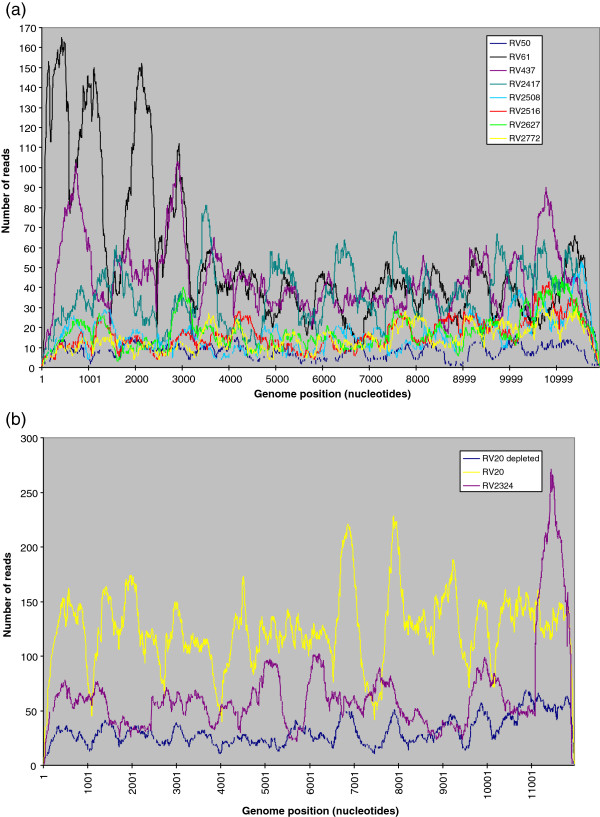
Depth of sequence obtained from (a) infected brain samples and (b) from cultured samples.

The depth of viral read coverage of samples from which a single genome contig was obtained was investigated (Figure 1). Brain tissue sample read depths varied from RV50, total viral reads 293, maximum read depth 16, and average read depth of 8.6; to RV437, total viral reads 1,489, maximum read depth 103, and average read depth of 44 (Figure 1a, Tables 2 and 3). RV61 did have a higher number of viral reads, but from two runs, therefore not directly comparable. Read depths from cell cultured viral samples were higher, with average read depths of 31 (RV20 depleted), 125 (RV20) and 65 (RV2324 cell pellet) and maximum read depths of 69 (RV20 depleted), 228 (RV20) and 272 (RV2324 cell pellet) (Figure 1b, Table 3).

**Table 3:** Details of single nucleotide polymorphisms (SNPs) detected within the 454 data

**Virus**	**Average depth**	**Maximum depth**	**Depth at SNP**	**% variant**	**Position (genome)**	**Gene (Position)**	**Nuc cons**	**Nuc variant**	**AA cons**	**AA variant**	**AA residue**
**Brain tissue samples**											
RV437	44	103	60	8	2768	M (275)	T	C	Leu	Ser	92
			63	3	2781	M (288)	T	C	Val	Val	96
			55	51	3049	M (556)	T	C	Leu	Phe	186
			28	18	4403	G (1090)	T	C	Leu	Leu	364
			28	7	7855	L (2451)	C	T	Asn	Asn	817
RV2417	35.5	81	NA								
RV61	53.2	165	34	18	3503	G (209)	C	T	Ile	Thr	70
RV2508	17	53	17	23	9523	L (4128)	A	G	Ser	Ser	1376
RV2627	17.7	46	NA								
RV2516	16.5	43	NA								
RV50	8.6	16	11	27	11349	L (5944)	C	G	Asn	Asp	1982
RV2772	14.5	31	13	38	1293	N (1223)	A	G	Lys	Arg	408
			22	5	8179	L (2771)	T	A	Phe	Tyr	924
**Cultured virus samples**											
RV2324	65	272	47	11	3490	G (175)	G	A	Glu	Lys	59
			47	21	3491	G (176)	A	G	Glu	Gly	59
			42	31	3711	G (396)	C	A	His	Gln	132
			44	25	4103	G (788)	A	G	Gln	Arg	263
			36	11	8376	L (2967)	C	T	Asp	Asp	989
RV1787 combined	4.3	11	4	25	2679	M (170)	G	A	Gly	Glu	57
RV20 combined	156	277	107	2	346	N (276)	C	A/T	Tyr	*/Tyr	92
			87	2	1621	P (105)	T	C	Ser	Ser	35
			81	2	1669	P (153)	G	A	Glu	Glu	51
			77	3	1710	P (194)	A	T	Gln	Leu	65
			93	39	3143	M-G (NA)	C	T	NA	NA	NA
			103	2	3671	G (356)	G	A	Trp	*	119
			96	2	4403	G (1087)	T	C	Cys	Arg	363
			94	37	4584	G (1268)	T	C	Met	Thr	423
			95	2	6217	L (766)	T	C	Ser	Pro	256
			134	1	7910	L (2459)	T	C	Ile	Thr	820
			107	2	8244	L (2793)	C	A	Ser	Arg	931
			106	2	8473	L (3022)	A	G	Thr	Ala	1008

NA = not applicable as no SNPs were observed. * stop codon.

### Viral heterogeneity analysis

**Figure 2:**
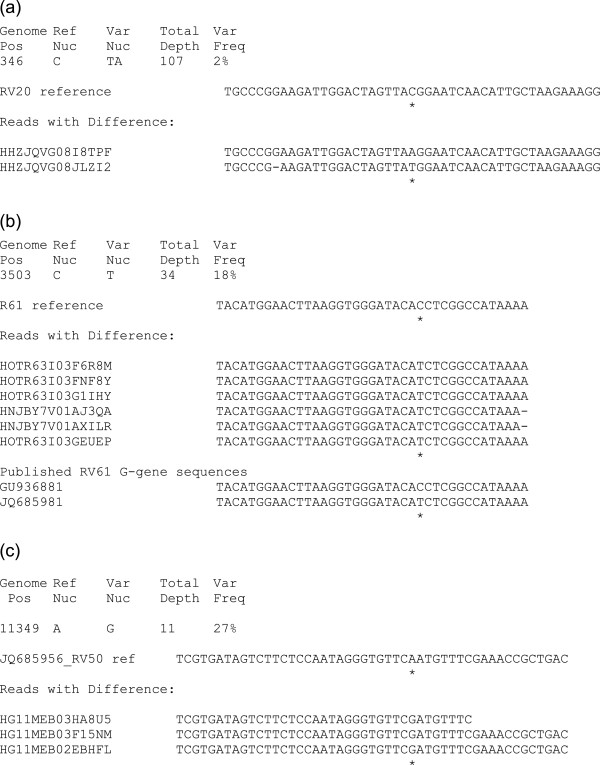
Alignment of 454 reads for (a) RV20 consensus sequence and the reads with variations, (b) RV61 consensus sequence and published RV61 glycoprotein gene sequences and (c) RV50 published genome sequence; * indicating single nucleotide polymorphisms which are detailed at the top of each figure.

Due to the processes involved, each read is obtained from a single cDNA strand, and therefore where multiple reads cover a region viral heterogeneity can be detected within the reads indicating the presence of a heterologous viral population. Although this methodology is not optimized for investigation of low level viral populations, which would require viral read depths of over 10,000, it is still possible to observe dominant or high level single nucleotide polymorphisms (SNPs). Even with this dataset, we were able to observe minimum SNPs (equating to one read with a SNP) at 1% of the population in cultured material, and 3% in brain tissue samples (Table 3). In general, cell cultured samples had more SNPs than tissue samples, where some tissue samples (RV2417, RV2627, RV2516) had no detectable SNPs (Table 3). Of the 28 nucleotide substitutions observed, 22 (79%) were conservative (pyrimidine to pyrimidine or purine to purine). Substitutions were identified throughout the genome, although only one was identified outside of a coding region (RV20 at position 3143 in the M-G untranslated region). At the amino acid level, 8 (29%) were synonymous substitutions resulting in no amino acid change (Table 3). Two stop codon substitutions were identified in this dataset, both in the RV20 tissue culture supernatant samples, and both at a low level (2%) equating to a single read (Table 3). Furthermore, one of these (N-gene position 276) had two reads which differed from the consensus, one encoding a stop codon, resulting in a truncated Nucleoprotein transcript, the other a synonymous change (Tyr/Tyr^92^) (Figure 2a).

RV61, an Arctic-like RABV, had one SNP detected in the 454 read data at genome position 3503 (Figure 2b). This SNP is located in the glycoprotein gene resulting in an amino acid substitution at position 70 Ile/Thr at a frequency of 18%. Analysis of currently available lyssavirus glycoprotein sequences, indicates this residue is Isoleucine in the majority of lyssavirus species. RV61 glycoprotein sequence is available in Genbank, deposited separately by two independent laboratories. No original material is available for analysis and the published sequences are from passaged viruses. Interestingly, alignments of the published sequences and the sequence obtained in this study are 100% identical, apart from this residue, where JQ685981 had Ile^70^ and GU936881 had Thr^70^ (Figure 2b).

RV50, a US isolate (isolated from *Eptesicus fuscus*), was genome sequenced using Sanger sequencing as part of a larger dataset [6], providing a unique opportunity to analyze the same virus isolate, propagated and sequenced independently. Alignments of the published RV50 genome (JQ685956) and the consensus sequence obtained in this study showed 100% identity. However, the 454 data identified a SNP at genome position 11,349 (L-gene), resulting in a non-synonymous coding change at residue 1982 resulting in conservative substitution Asn/Asp^1982^ (Figure 2c). The frequency of this variant in the read data was 27% (Table 3).

RV2508 was sequenced both from the host brain tissue directly and from cell culture supernatant after 6 passages in BHK cells. Only one SNP was observed at position 9523 (L-gene) a synonymous coding change A/G^9523^. Of the 17 original brain tissue reads covering this region, 23% were G^9523^, whereas the tissue culture supernatant sample reads (n = 2) only had G^9523^. To investigate this variation further, specific primers were designed to amplify this region from the original brain sample and the passaged TCSN. The original brain material PCR amplicon had a read depth of 3783 reads, 18.4% G^9523^ and 81.6% A^9523^. The TCSN PCR amplicon had a read depth of 3280 reads, 97.5% G^9523^ and 2.5% A^9523^, confirming the consensus sequence data, but indicating the A variant is still present after 6 passages, just at a much lower level. This deep sequence analysis is a useful tool to investigate certain SNPs of interest indicated from the consensus data.

RV437, a raccoon-dog RABV from Estonia, had good coverage across the genome (average read depth 44, maximum read depth 103) and also had the most SNPs observed within a tissue sample (Table 3). Across the genome, 5 SNPs were detected, 3 within the M-gene (2 of which resulted in amino acid changes – Leu/Ser^92^ and Leu/Phe^186^) and one synonymous change at genome position 2781. The remaining two were both silent, one in the glycoprotein gene (genome position 4403) and the other in the L-gene (genome position 7855) (Table 3).

Although a full genome contig was not obtained for RV1787, 6 contigs covering approximately 90% of the genome were generated. Full genome sequence had previously been generated using overlapping PCR products and Sanger sequencing [1]. Comparison between the Sanger and 454 generated sequences revealed 100% match apart from a single nucleotide polymorphism (SNP) at genome position 2679 (residue 57 in the Matrix Protein) which corresponded to substitution Gly/Glu (Table 3). Published sequences of all lyssavirus species (including the recently identified IKOV; [22] have Gly^57^. The read coverage at this position was 6 reads, 5 of which contained the Glu^57^ SNP and 1 contained the original Gly^57^, present in the Sanger sequencing and all other lyssaviruses.

### Obtaining genomic termini

The genomic termini sequences from 454 data were obtained with varying success. The 5′ UTR was usually represented by reads more often than the 3′ UTR, the reasons for which are unclear (Table 2 and Figure 1). Often *de novo* assembly failed to incorporate reads which contained the genomic termini. These reads were only incorporated after splicing the missing sequence, from a published similar genomic sequence, to the consensus sequence deduced from the *de novo* assembly and subsequently mapping reads against the spliced reference using GS mapper. Subsequent to this dataset, in an attempt to increase the population of genomic end viral reads, the 454 sample preparation methodology was modified by the addition of 1pmol of N165-146 and LRACEF2 at the hexamer cDNA synthesis stage, to enhance the cDNA population containing the genomic termini. This modification was trialed on a genome sample highly related to RV2772, where the genomic ends were obtained (data not shown). This simple modification will be used for future genome sequences.

For samples where the genomic termini were still absent, RT-PCR was performed on the depleted RNA, using primers designed against the highly conserved genomic termini to obtain PCR products which were sequenced directly using Sanger sequencing. Although this approach resulted in a genome sequence still lacking the sequencing relating to the primer sites, for known lyssavirus species isolates this is acceptable. However, for novel lyssavirus species prototypes the ends must be deduced either by RACE [1] or circularization of the RNA, followed by RT-PCR [2]. To this end IKOV genomic ends were obtained using circularization of the genomic RNA [22].

### Confirming correct sequence and differentiating between indels/homopolymeric repeats

**Figure 3:**
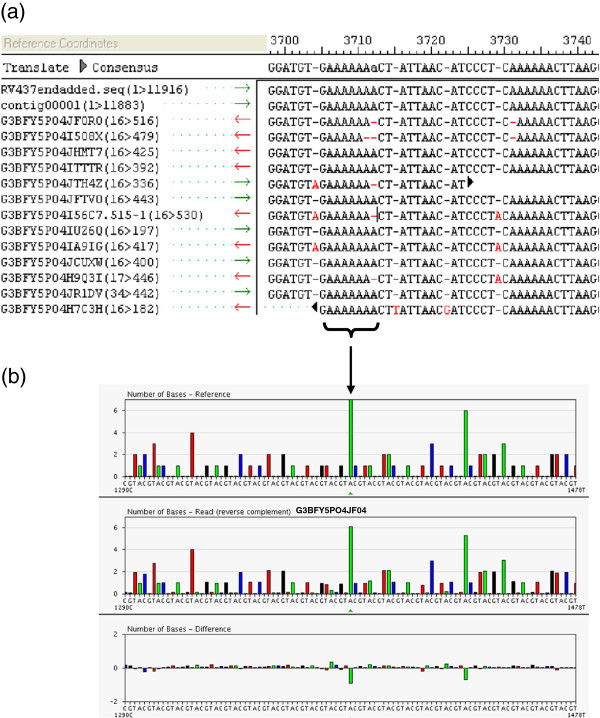
Typical variation of the reads over a homopolymeric repeat at the termination signal of the M-gene displayed as (a) an alignment of individual 454 reads with the consensus sequence (contig00001) and a reference sequence (RV437) and (b) a flowgram with reference sequence (RV437, top panel), read G3FY5P04JF0R0 (middle panel) and the difference between the reference sequence and read (bottom panel).

Whenever possible, consensus sequences where aligned together with genome sequences from the same lyssavirus species. Any potential insertions or deletions (indels) were investigated by analysis of the flowgrams in the GS program suite. A limitation of pyrosequencing processing of raw data is runs of homopolymers such as the GAAAAAAA poly A termination signal found at the end of each lyssavirus gene sequence (Figure 3a and b). Figure 3 illustrates the discrepancies between individual reads from the same sample in a homopolymeric run, where the signal strength for each base is plotted for each read. Detecting the correct signal in a homopolymer of four or more bases can be problematic, resulting in incorrect base calling. In general, the more reads covering the genome the more accurate the resulting consensus sequence (represented by contig00001 in Figure 3a). Where discrepancies cannot be resolved, or a new virus species is being sequenced, confirmation of the correct sequence using PCR products spanning the region in question sequenced using Sanger technologies may be required. In this dataset, only the proposed new lyssavirus species IKOV (RV2508) required additional PCR confirmation. Each intergenic region length and sequence was confirmed by RT-PCR, with 100% concurrence between the 454 consensus sequence and the Sanger sequence derived from PCR products (data not shown).

## Discussion

Our goal was to establish a methodology which enabled sequencing from brain tissue, and cell cultured samples without the use of specific viral enhancement, ultracentrifugation or filtration to identify novel viruses, or obtain genome sequence for known virus species. It was important to develop a methodology which would not require prior knowledge of the virus being extracted and to use a methodology which is widely used to extract viral RNA. Instead of enriching for virus specific RNA, we depleted host genomic DNA and rRNA to increase the percentage of viral specific RNA in the sample. There are a large number of RNA extraction methodologies and kits available which can be broadly divided into two categories; 1) Phenol/choloform based extraction and 2) column or bead based extraction. We decided to compare TRIzol® as a phenol-based system and RNeasy® plus mini kit (Qiagen) as a column-based system. In our hands, the use of spin columns to extract the RNA from tissue, removed the majority of RNA in process, resulting in less than the 200 ng minimum quantity of total RNA recommended for fragmentation and more importantly, too few viral reads were obtained. The RNeasy® plus mini kit (Qiagen) was successfully used as part of the depletion protocol after initial extraction with TRIzol® with good results. In this study, less than 200 ng of RNA was used for a number of samples, and depending on the sample type less than 200 ng of RNA can provide full genome sequence. TRIzol® extracted tissue samples RV50 (153 ng RNA) and RV2772 (30 ng RNA), not only obtained sufficient reads to cover the genome, but also obtained enough depth of coverage to identify SNPs (Tables 2 and 3). Furthermore, for RV20 depleted tissue culture supernatant only 10 ng of RNA was fragmented, yet 1,054 viral specific reads were mapped equating to 41% of the total reads obtained. These observations are important to illustrate that the methods for library preparation can be further refined for virus sequencing.

The requirement to deplete tissue culture supernatant derived RNA was investigated using two 100 ml supernatant samples. Half the RNA was subjected to gDNA and rRNA depletion, whereas the other was not. The additional preparation time required to deplete the sample was not rewarded with a significant improvement of either proportion of viral-specific reads or read depth, therefore 454 sequencing directly from a TRIzol® extracted RNA is the simplest, yet most effective way to obtain viral genome sequence from PEG precipitated tissue culture supernatant samples. However, it is not always possible, or practical to culture viruses *in vitro*. Furthermore, any passaging of viruses in a cell culture system will apply selective pressures on the virus and, depending on the number of passages necessary to obtain virus at a high enough titer to harvest, will alter the population of virus being sequenced. Indeed, the number of SNPs observed in the cell cultured RNA preparations were greater than the tissue extracted samples (Table 3). Furthermore, the number of non-conservative substitutions observed (both nucleotide and amino acid) was significantly more in the cultured samples, including the presence of stop codons (Table 3). The RV2508 synonymous SNPs identified between the brain material and the TCSN passaged 6 times in BHK was investigated by designing specific primers to amplify across the SNP from both the original and cultured virus samples. ‘Deep sequencing’ of this amplicon determined that the dominant viral population changed from G^9523^ to A^9523^ after 6 passages, and although no A^9523^ SNP reads were observed in the TCSN consensus sequencing they were still present in the population, just at a low level (2.5%). Although for this example there is no functional relevance for the variation, this approach is useful to determine high proportion SNP changes within passaged virus samples using consensus sequencing which can then be targeted directly by deep sequencing the relevant genomic regions, rather than deep sequencing the entire genome.

The genomic termini are often under-represented in the viral reads obtained. It is likely this is due to a variety of reasons including the process of trimming reads before assembly, the assembly parameters in *de novo* and mapping programmes as well as biological influences such as mRNA over-representing the viral RNA population relative to genomic RNA and the effect of defective interfering (DI) particles which are often truncated. The use of primers situated near the genomic termini during the cDNA synthesis stages have been shown to improve the number of termini sequences, but have not completely resolved the problem.

Sequences generated on the 454 platform, due to the way the incorporation of bases are detected by the intensity of light emission, inherently have issues with long homopolymeric repeats. On the whole, a consensus sequence with 10 or more reads, when mapped using an available genome from the same species, can be confidently confirmed by checking the flowgram data. However, novel viruses, or viruses with a limited number of reads may require additional confirmation by Sanger sequencing PCR products, which span the regions in question.

## Conclusion

The application of this methodology to lyssaviruses from brain material and cell cultured samples has been shown to be highly successful. Moreover, we have successfully sequenced the majority of a flavivirus and hantavirus, demonstrating the applicability of the method to other families of viruses (data not shown). There is no reason why any cultured virus cannot be PEG precipitated, extracted and sequenced using the same methodology. We have shown for both a high titer virus (RV20) and a low titer virus (RV1787) 100 ml of supernatant is more than enough to obtain genome sequence, and to begin to investigate the presence of viral heterogeneity. Further investigations using 5-10 ml of tissue culture supernatant for a number of other cultured lyssaviruses have proved equally as successful (data not shown). Furthermore, extraction from the cell monolayer (often discarded when clarifying harvested supernatant) is an excellent source of viral RNA. The only disadvantage is the potential presence of non-functional virus variants presumably present in the cells as partially synthesized viral derivatives. In this study however, it was virus in the supernatant which appeared to contain defective virion particles, albeit at a low level.

The platform used in this study (Roche 454) has been shown to be reliable and consistent, however, the RNA can be prepared for any NGS platform. Indeed we have obtained full genome sequences from a small number of RNA samples prepared as described here from brain material on the Illumina platform successfully (data not shown). The application of NGS sequencing of viruses has begun a new era of virus discovery and characterization of novel viruses and will revolutionize phylogeographic studies in all fields of virology in the coming years.

## Methods

### Virus samples

Original clinical specimens, or once passaged mouse brain samples (Table 1), confirmed positive previously by FAT (Fluorescent Antibody Test) and by RT-PCR were used. Cultured viruses were passaged from original brain samples in Baby Hamster Kidney cells. All in vivo work was undertaken in BSL3/SAPO4 containment in AHVLA, following independent ethical review and complied with the Animal Scientific Procedures Act 1986.

### PEG precipitation

Concentration of virus in tissue culture supernatant (TCSN) over 1 ml (specifically RV20 and RV1787) was achieved using 1 volume of PEG-it (System Biosciences) to 4 volumes of TCSN and following manufacturer’s instructions. Pellets were combined and resuspended in tissue culture media to a final volume of 1 ml.

### RNA extraction

TCSN samples were clarified to remove cell debris by centrifugation at 1,200 rpm for 5 mins and were extracted either directly (250 μl) or after PEG precipitation (see above) using TRIzol® LS following manufacturer’s instructions and resuspended in 10 μl molecular grade water. All other samples were extracted using TRIzol® following manufacturer’s instructions and eluting in 10 μl molecular grade water. For RNA extraction method comparisons, duplicate samples were extracted using RNeasy® plus mini kit (Qiagen), including the DNase treatment (see 2.4).

### gDNA depletion

Genomic host DNA was depleted from the extracted RNA samples using the on-column DNase treatment in RNeasy® plus mini kit (Qiagen) following manufacturer’s instructions, eluting in 30 μl molecular grade water.

### rRNA depletion

Ribosomal RNA was depleted from the gDNA-depleted RNA samples using Terminator™ 5′-Phosphate-Dependent Exonuclease (Epicentre Biotechnologies). The reaction was performed according to the manufacturer’s instructions, briefly, 30 μl of RNA was mixed with 3 μl of Buffer A, 0.5 μl RNAsin® Ribonuclease inhibitor (20–40 U/μl, Promega) and 1 μl Terminator (1 U/μl). The mixture was incubated at 30°C for 60 minutes. Thereafter the samples were subjected to subsequent round of purification using RNeasy® plus mini kit (Qiagen), without DNase digestion, following manufacturer’s instructions, eluting in 30 μl molecular grade water.

### RNA quantification and purity check

After extraction and then gDNA depletion, the RNA was quantified using NanoDrop® spectrophotometer (results in Table 2). After rRNA depletion the final RNA sample was quantified using RiboGreen® on a spectrophotometer before commencing the 454 RNA preparation protocols. For TCSN samples which did not undergo depletion, the RNA was diluted to 1000 ng/ul according to the nanodrop concentration, then quantified using RiboGreen® on a spectrophotometer.

### RNA fragmentation, cDNA library amplification and sequencing

200 ng of RNA was fragmented using divalent cations (ZnCl_2_) at 70°C for 30 s. Where less than 200 ng was available the maximum volume (20 μl) was used. The fragmented RNA was then purified using RNAclean XP (Beckman Coulter) magnetic beads and used as template for double-stranded cDNA synthesis using random hexamers (Roche) and a cDNA Synthesis System Kit (Roche) according to the manufacturer’s instructions. To improve the population of viral reads at the genomic termini, two panlyssavirus specific primers were included in the random RT reaction: N165-146 (GCAGGGTAYTTRTACTCATA) previously described [25] and LRACEF2 (TGAGTCTRTCATCTCACTGG) at 1 pmol/μl. Ends were repaired and specific sequencing adapters with multiplex identifiers ligated using the Rapid Library Kit (Roche). The resultant fragments were purified and size-selected using Ampure XP (Beckman Coulter) magnetic beads and the libraries quantified with High Sensitivity DNA chips on a BioAnalyzer (Agilent).

Libraries were pooled as appropriate in equimolar concentrations. Pooled fragments were clonally amplified using emPCR kits (Roche) and sequenced on the Roche 454 GS FLX + instrument according to the manufacturer’s instructions. Each sample was loaded onto 16th picotitre 454 plate.

### Sequence data analysis

Initial assembly of the 454 reads was achieved with Newbler (V2.6) using the GS *de novo* assembly software (Roche). All contigs were exported from the software and were aligned with a reference genome using Seqman (DNAStar) whenever possible. The resulting consensus sequence was subsequently used in GS Reference Mapper (Roche) to obtain further sequence reads from the original raw data. If a suitable reference sequence was available, this was used in the GS Reference Mapper program with the 454 reads to obtain a consensus sequence without the initial use of the GS *de novo* assembly software. Where no suitable genome reference sequence was available (RV2508) a BLAST search with the contigs provided evidence for which contigs were viral sequence, using the megablast algorithm on the nucleotide database (http://www.ncbi.nlm.nih.gov/blast/Blast.cgi?CMD=Web&PAGE_TYPE=BlastHome). Due to the increased difficulty of mapping reads at the ends of contigs, it was occasionally necessary to splice the 3′ and/or 5′ UTR sequences from a reference genome sequence onto the termini of the contig sequence of interest. Using the spliced consensus sequence in the GS Reference Mapper program resulted in extra reads being mapped to the contig resulting in a complete, or almost complete genome sequence.

### Obtaining genomic termini

The lyssavirus genomic termini are extremely conserved, in particular the first and last 9 nucleotides have complete complementarity across all lyssaviruses [1,2,5,22], furthermore the conservation remains stringent until residue 25 [2]. Previously described panlyssavirus primers situated at the leader and trailer extremities, leader: LYS001F (5′ACGCTTAACGAMAAA3′) and trailer: LYSEND (5′ACGCTTAACAAAWAAA3′) [2] were used with panlyssavirus primers JW6UNI (5′CARTTVGCRCACATYTTRT3′) and LgeneFor (5′CTCACTGGATMAGRTTRATITACAA3′) respectively to obtain PCR products which were sequenced directly using the PCR primers as described elsewhere [26].

### SNP ‘deep sequencing’

Primers flanking the SNP identified for IKOV: RV2508-L-SNP-F (5′ CTGAAGCTTCGAGACTCTAC 3′) and RV2508-L-SNP-F (5′ CAGATGGATGACCCTATCAG 3′) were used in a standard RT-PCR reaction, using high fidelity enzymes in methods described previously [1]. The amplicons were purified using Ampure XP (Beckman Coulter) magnetic beads and specific sequencing adapters with multiplex identifiers were ligated using the Rapid Library Kit (Roche) and purified using Ampure XP (Beckman Coulter) magnetic beads. The purified ligated amplicons were size selected and quantified with High Sensitivity DNA chips on a BioAnalyzer (Agilent). Amplicons were pooled as appropriate in equimolar concentrations and clonally amplified using emPCR kits (Roche) and sequenced on the Roche 454 GS FLX + instrument according to the manufacturer’s instructions. The RV2508 genome sequence was used in the GS Reference Mapper program with the 454 reads to determine the percentage of reads present for the SNP.

## Abbreviations

NGS: Next generation sequencing; BHK: Baby hamster kidney; SNP: Single nucleotide polymorphism.

## Competing interests

The authors declare they have no competing interests.

## Authors’ contributions

Funding was obtained by DM, LM, XDEL and ARF. DM carried out sequence analyses. DM, EW, SL carried out and optimised the experiments. DM, LM, RE, DH, XDEL and ARF designed the study. DD and RE provided reagents and tools. DM drafted the manuscript. All authors contributed to and approved the final manuscript.
